# A spatial map: a propitious choice for constraining the binding problem

**DOI:** 10.3389/fncom.2024.1397819

**Published:** 2024-07-02

**Authors:** Zhixian Han, Anne B. Sereno

**Affiliations:** ^1^Department of Psychological Sciences, Purdue University, West Lafayette, IN, United States; ^2^Weldon School of Biomedical Engineering, Purdue University, West Lafayette, IN, United States; ^3^Department of Family Medicine, Indiana University School of Medicine, Indianapolis, IN, United States

**Keywords:** retinotopic, cortical maps, two-streams hypothesis, feature integration, visual perception, convolutional neural network, deep learning

## Abstract

Many studies have shown that the human visual system has two major functionally distinct cortical visual pathways: a ventral pathway, thought to be important for object recognition, and a dorsal pathway, thought to be important for spatial cognition. According to our and others previous studies, artificial neural networks with two segregated pathways can determine objects' identities and locations more accurately and efficiently than one-pathway artificial neural networks. In addition, we showed that these two segregated artificial cortical visual pathways can each process identity and spatial information of visual objects independently and differently. However, when using such networks to process multiple objects' identities and locations, a binding problem arises because the networks may not associate each object's identity with its location correctly. In a previous study, we constrained the binding problem by training the artificial identity pathway to retain relative location information of objects. This design uses a location map to constrain the binding problem. One limitation of that study was that we only considered two attributes of our objects (identity and location) and only one possible map (location) for binding. However, typically the brain needs to process and bind many attributes of an object, and any of these attributes could be used to constrain the binding problem. In our current study, using visual objects with multiple attributes (identity, luminance, orientation, and location) that need to be recognized, we tried to find the best map (among an identity map, a luminance map, an orientation map, or a location map) to constrain the binding problem. We found that in our experimental simulations, when visual attributes are independent of each other, a location map is always a better choice than the other kinds of maps examined for constraining the binding problem. Our findings agree with previous neurophysiological findings that show that the organization or map in many visual cortical areas is primarily retinotopic or spatial.

## Introduction

Many neuropsychological, lesion, and anatomical studies have shown that the human visual system has two major functionally distinct cortical visual pathways (Ungerleider and Mishkin, 1982; Mishkin et al., 1983; Felleman and Essen, 1991): a ventral pathway, thought to be important for object recognition (Logothetis and Sheinberg, 1996), and a dorsal pathway, thought to be important for spatial cognition (Colby and Goldberg, 1999). Many studies have shown that artificial neural networks with two segregated pathways (e.g., identity and location) can achieve higher performance in visual tasks than artificial neural networks with a single pathway (Rueckl et al., 1989; Scholte et al., 2018; Han and Sereno, 2022). Specifically, artificial neural networks with two segregated pathways can identify and localize objects with higher accuracy and efficiency than single-pathway neural networks (Han and Sereno, 2023a). However, when there is more than one object in a visual image, the binding problem can arise because the network may not be able to correctly associate each object's identity with its location (Feldman, 2013; Greff et al., 2020). The binding problem is one of the fundamental challenges in the field of artificial intelligence because it prevents artificial neural networks from forming a compositional understanding of the world, which is crucial for symbolic reasoning and human-level generalization (Greff et al., 2020; Zheng et al., 2022).

Some previous studies proposed that the binding problem could be solved using temporal synchrony binding using spike timing information of neurons (Milner, 1974; von der Malsburg, 1999; Zheng et al., 2022). However, some studies failed to find experimental evidence that supports the binding by synchrony model (Dong et al., 2008). Other studies found that when animals were performing perceptual tasks related to binding, the behaviorally relevant information was carried by firing rates, and spiking synchrony carried no information (Shadlen and Movshon, 1999). Therefore, here, we explore constraining the binding problem in the other ways. In our previous study, we constrained the binding problem by retaining relative location information of objects while training an artificial identity pathway in a two-pathway network (Han and Sereno, 2023a). As a result, the differently and independently retained location information in the two pathways could be used to bind each object's identity with its absolute (not relative) location. This solution may be considered as using a relative location map to constrain the binding problem because both the identity pathway (relative locations) and the location pathway (absolute locations) were trained to report the results according to objects' locations. However, this previous study only considered two attributes (identity and absolute location) of objects and only one possible constraint map (relative location map), but ordinarily there may be other attributes to process and bind, and any of these possible attributes could be used as the constraint map. In our current study, we generalized our study to include four attributes of objects: identity, luminance, orientation, and location. Furthermore, under these conditions and constraints, we looked for the best map(s) to constrain the binding problem when different pairs of these attributes are combined. Any usage or claim of “best” or “optimal” or “always” is limited to the conditions and simulations we have conducted in our current study. Because our previous studies have shown that two-pathway networks are better than one-pathway networks when we need to bind or conjoin two attributes of each object at the same time, we always used two-pathway networks in our current study. Since our previous study also showed that it was possible to bind each object's identity with its absolute location with the help of a relative location map, we want to find out whether it is also possible to use a similar method with other attributes to constrain the binding problem (e.g., relative identity map) when pairs of attributes are being bound. Theoretically, there could be several different choices for using a map to constrain the binding problem with two attributes. For example, we could use a relative location map to constrain the binding problem (as we did originally), or we could use a relative identity, luminance, or orientation map to constrain the binding problem. Preliminary modeling results for our given conditions and constraints suggest that even though it is possible to constrain the binding problem using different kinds of maps, a location map will always achieve higher accuracy than an identity, luminance, or orientation map, even when neither network pathway is trained to recognize location (Han and Sereno, 2023b).

In this study, we used feed-forward convolutional neural networks to simulate different visual pathways. All neural networks are trained using supervised learning, and all artificial visual pathways have the same structure and size. We use stochastic gradient descent with back-propagation to update the weights in the neural networks during training. We train the different artificial visual pathways (branches) separately with different goals so that they will be specialized to recognize different attributes of objects (including identity, luminance, orientation, or location). We use two-pathway networks to simultaneously determine any two of these visual attributes of the objects, where each pathway in the two-pathway network is pre-trained to determine one attribute of the objects.

**Figure 1:**
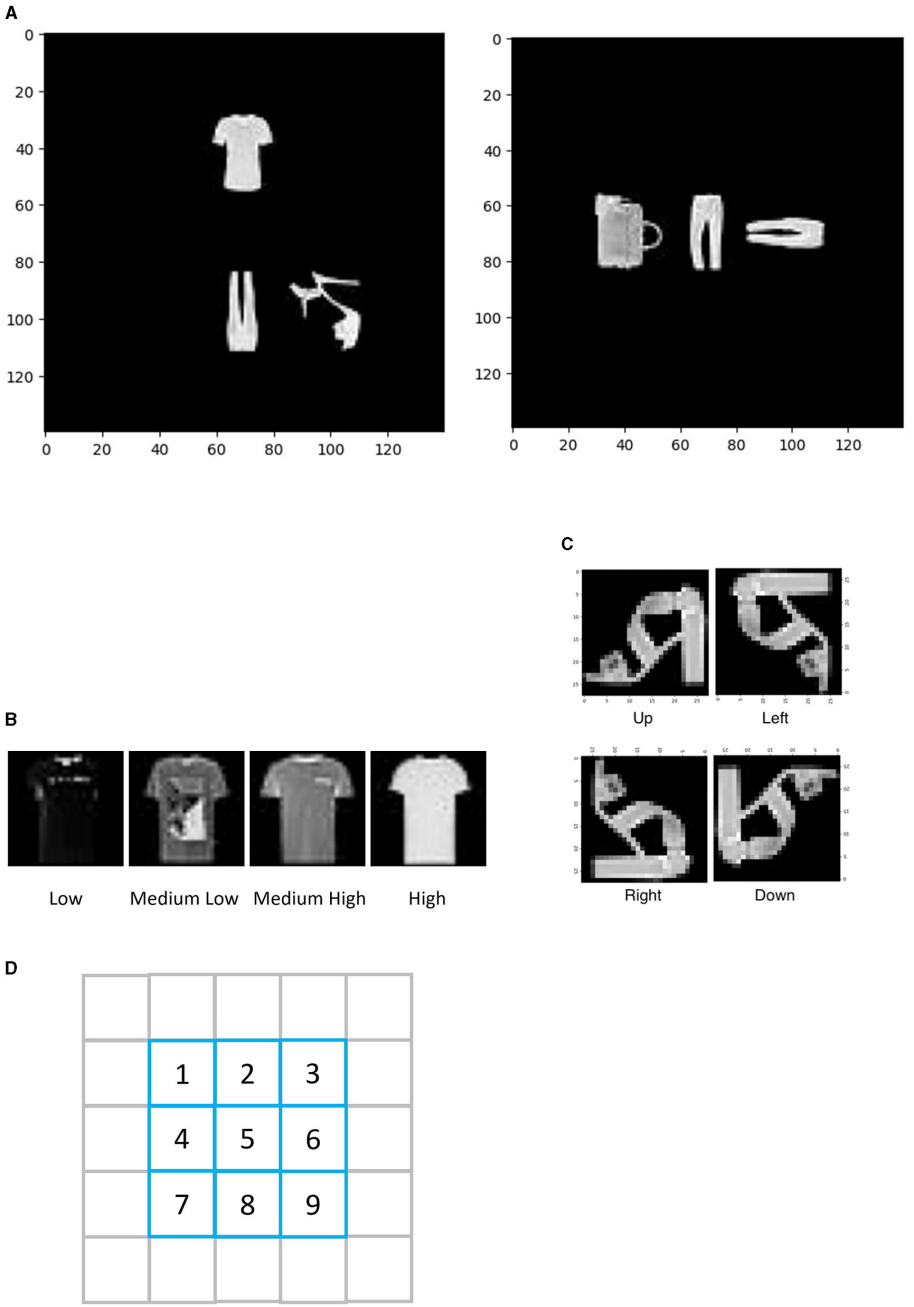
Explanations of visual images. **(A)** Two examples of visual images. **(B)** An example of a t-shirt with low luminance, a t-shirt with medium low luminance, a t-shirt with medium high luminance, and a t-shirt with high luminance. **(C)** An example of a shoe with orientations up, left, down, and right. **(D)** Nine possible locations of the objects in the visual image.

Black and white images consisting of different kinds of shirts, pants, shoes, and bags are used as the objects in the images (see Figure 1). Multiple objects are randomly selected and put in front of a black background at one of the nine possible locations. These images with multiple objects are used as input images to the neural networks (see Methods section for details). We chose to use relatively simple images because it is easier to conduct controlled experiments and make comparisons between the many different models.

According to our simulations, using the location map to constrain the binding problem is able to achieve the highest accuracy in most cases, but there are exceptions. The exceptions occur when there might be interactions between different visual attributes. According to our simulations, when the dependency between different attributes is removed, using the location map to constrain the binding problem (for any two of our four attributes) in these experiments always achieves a higher accuracy than using another map (identity, luminance, or orientation). In addition, when different attributes are independent, the advantage of using the location map is unrelated to the number of classes in an attribute or whether the objects share the same value in an attribute.

Our results agree with experimental evidence that shows the visual system in our brain is primarily using spatial maps to encode different attributes of objects (Kaas, 1997; Lennie, 1998). Our results also generally agree with some previous theories about the binding problem that propose the brain is using space to bind different attributes together (Treisman, 1998). However, in our study, we used local (distributed) and relative location maps, not a master and absolute location map that was proposed in Treisman (1998). Topographic maps in sensory cortex are widely documented. However, the functional roles of the topographic maps are still unclear (Kaas, 1997; Weinberg, 1997; Arcaro and Livingstone, 2021). According to our study, we speculate that one functional consequence of topographic maps in the visual system is the efficient binding of various visual attributes and the ability to form integrated objects.

## Methods

### Objects and visual images

Black and white images of different kinds of t-shirts, pants, shoes, and bags obtained from the data set Fashion-MNIST were used as the objects in the visual tasks (Xiao et al., 2017). There are 200 kinds of t-shirts, 200 kinds of pants, 200 kinds of shoes, and 200 kinds of bags. To make sure that the orientations of the bags were clearly defined, only bags with handles were selected to be used in the visual tasks. Some examples of the objects are shown in Figure 1. Each object was embedded in a black background and randomly presented at different locations in a 140 × 140 (pixels) black square background. There are three objects in each black background visual image. These visual images with objects and black background were used as visual inputs. Two examples of these visual images are shown in Figure 1A. Six thousand visual images were randomly created unless stated otherwise. Two-thirds of these visual images were used as training data, one-sixth of them were used as validation data, and one-sixth of them were used as testing data.

### Four attributes of objects

In this study, we considered four attributes of objects: identity, luminance, orientation, and location.

#### Identity

There are four kinds of object identities: t-shirt, pant, shoe, and bag. An example of an visual image containing a t-shirt, a pant, and a shoe is shown in Figure 1A, left panel. An example of an visual image containing a bag and two pants is shown in Figure 1A, right panel.

#### Luminance

The luminance value of each object is defined as the average value of all the pixels in the object image. The maximum possible value of each pixel is 255 (white), and the minimum possible value of each pixel is 0 (black). Note there are some black background pixels within each object image (as shown in Figures 1B, C), and these black background pixels are also included when calculating the object's luminance value. Each object's luminance value is then classified into one of four luminance levels: high (luminance value ≥ 3 * 255/8), medium high (2 * 255/8 ≤ luminance value < 3 * 255/8), medium low (255/8 ≤ luminance value < 2 * 255/8), and low (luminance value < 255/8). Examples of different luminance levels are shown in Figure 1B.

#### Orientation

Each object has four possible orientations: up, left, down, and right. An example object in different orientations is shown in Figure 1C.

#### Location

Object locations are shown in Figure 1D. Specifically, each object can be centered at one of nine possible locations (locations numbered from 1 to 9; see Figure 1D). The objects in a given visual image never overlapped with each other.

### Neural networks

The different visual pathways in the brain were modeled using feed-forward convolutional neural networks. All neural networks were implemented using Tensorflow and were trained using supervised learning, the cross-entropy loss function, gradient descent with back-propagation, and Adam optimization method. Each neural network consists of convolutional layers, pooling layers, and fully connected dense layers. A ReLU activation function was used at each layer except the final output layer, in which a softmax activation function was used to output the classification results. The artificial neural networks in this study have structures similar to the networks that we used in our previous studies (Han and Sereno, 2022, 2023a).

**Figure 2:**
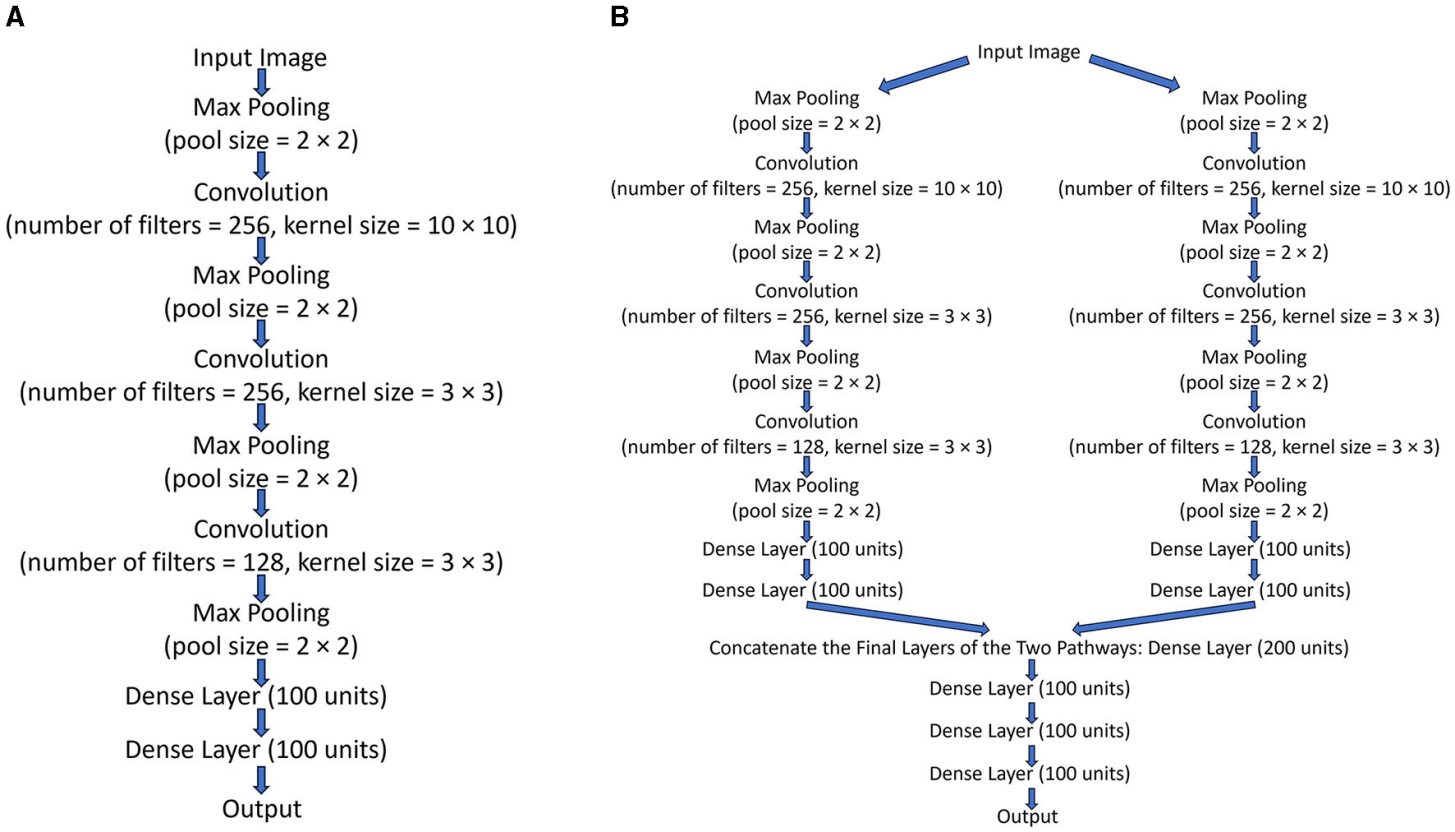
**(A)** The structure of *Network*_*identity*_, *Network*_*luminance*_, *Network*_*orientation*_, and *Network*_*location*_. **(B)** The structure of *Network*_*twopathways*_.

A batch size of 256 was used while training, and the initial learning rate of Adam optimization was 0.001. The other hyperparameters are specified in Figure 2. A 30% random dropout was applied to the dense layers in all neural networks during training for regularization. All networks were trained until they had reached the highest possible validation accuracy.

The structure of neural networks used to model different visual pathways is shown in Figure 2A. All of the neural networks used to model different visual pathways share the same structure, with the only difference between them being their final output layer. All visual pathway networks take the same set of visual images as inputs but are trained to do one of four different tasks, so their output layers have different sizes. These visual pathway neural networks were trained and then served as one of the two pathways in a two-pathway network (*Network*_*twopathways*_) to simultaneously determine any two attributes of all the objects in the visual images. The four visual pathway networks were trained to determine the four different attributes of objects: identity (*Network*_*identity*_), luminance (*Network*_*luminance*_), orientation (*Network*_*orientation*_), and location (*Network*_*location*_). These visual pathway networks were trained in different ways when different kinds of maps were used to constrain the binding problem. The binding problem could be constrained using the location map, the identity map, the luminance map, or the orientation map.

The structure of *Network*_*twopathways*_ is shown in Figure 2B. *Network*_*twopathways*_ was trained to determine two attributes of all the objects in each image simultaneously by processing the visual image in two segregated pathways and then combining them together. *Network*_*twopathways*_ took the visual images as inputs and sent this information into the two pathways. The two independently pre-trained visual pathway networks (excluding their output layers and with their weights fixed) were used as the two pathways in *Network*_*twopathways*_. The two segregated pathways processed the input images separately; then, the network concatenated the final layers of the two pathways together and processed the information jointly with some additional common dense layers. In sum, after the two pathways had been independently trained and their weights fixed, the common dense layers in *Network*_*twopathways*_ were trained to report the two attributes of all objects using one-hot encoding.

When the location map was used to constrain the binding problem and *Network*_*twopathways*_ needed to recognize identities and locations of all objects, the identity pathway was trained to report all objects' identities in a certain order that depended on the relative locations of the objects. Specifically, it was trained to report the identities of the objects at the top first. If objects were at the same horizontal line, then the network needed to report the identities of the objects from left to right. The specific order described here is an assumption without loss of generality: that is, any consistent order would suffice. The location pathway was trained to report all objects' absolute locations regardless of their other attributes. After training, both the identity pathway and the location pathway should retain some location information of objects and this location information can be used to constrain the binding problem. An important difference between the location information in the identity pathway and the location information in the location pathway is that the identity pathway was trained to retain the relative location information of objects (which object is at the top left relative to other objects), but the location pathway was trained to retain the absolute location information of objects (locations numbered from 1 to 9).

On the other hand, when the identity map was used to constrain the binding problem and *Network*_*twopathways*_ needed to recognize identities and locations of all objects, the identity pathway was trained to report all objects' absolute identities (i.e., t-shirts, pants, shoes, and bags). The location pathway was trained to report all objects' absolute locations in a certain order that depended on the relative identities of the objects. Specifically, the location pathway was trained to report the locations of the t-shirts first, then report the locations of the pants, then report the locations of the shoes, and finally report the locations of the bags. If two objects had the same identity (e.g., see Figure 1A, right panel), and then, the location pathway was trained to report the locations of them in any order. The specific order described here is also an assumption without loss of generality: that is, any consistent identity order would suffice (e.g., reporting the locations in the order of locations of bags, locations of pants, locations of t-shirts, and locations of shoes would also work).

It is important to note that for the example we have been using (*Network*_*twopathways*_ needing to recognize identities and locations of all objects), it is also possible to use a third map (the luminance map or the orientation map) to constrain the binding problem. In this case, we train the identity and location pathways to report objects' identities and locations in a certain order that depends on the relative luminance or relative orientations of the objects. Specifically, when the luminance map is used, we train the identity and location pathways to report the identities and locations of objects, respectively, with low luminance first, then report the identities and locations of objects with medium low luminance, then report the identities and locations of objects with medium high luminance, and finally report the identities and locations of objects with high luminance. This specific order is again an assumption without loss of generality: that is, any other consistent order would also suffice (e.g., reporting identities and locations of objects in the order of high, medium high, medium low, and low luminance would also work). Finally, when the orientation map was used for the example we have been using (*Network*_*twopathways*_ needing to recognize identities and locations of all objects), we trained the identity and location pathways to report the identities and locations of objects with orientation up first, then report the identities and locations of objects with orientation left, then report the identities and locations of objects orientation down, and finally report the identities and locations of objects with orientation right. This specific order is again an assumption without loss of generality: that is, any other consistent order would also suffice (e.g., reporting identities and locations of objects in the order of up, right, down, and left would also work).

Importantly, any third map may be used to constrain the binding problem for any other *Network*_*twopathways*_ trained to determine any other two attributes. For example, it is possible to use the location map when *Network*_*twopathways*_ needed to recognize luminance and orientations of all objects. In this case, we trained the luminance pathway and the orientation pathway to report objects' luminance and orientations in a certain order that depended on the relative locations of objects. Likewise, we also used the identity map, the luminance map, and the orientation map to constrain the binding problem. In sum, we trained *Network*_*twopathways*_ to determine all possible pairs of attributes with all different kinds of maps as the constraint.

Each visual pathway and two-pathway network was trained three times, and the testing accuracy from each of the three training sessions was recorded. The testing accuracy was obtained by dividing the number of correct classifications by the total number of testing samples during the testing session. The accuracies that are used to compare different networks in this study are always referring to the testing accuracies. The percentage gains of accuracy for different tasks when the location map as opposed to the identity map, the luminance map, or the orientation map was calculated by $Gain=\frac{Location\;Map\;Accuracy-Other\;Map\;Accuracy}{Other\;Map\;Accuracy}\times 100\%$. Where *LocationMapAccuracy* is the average *Network*_*twopathways*_ accuracy obtained using the location map, *OtherMapAccuracy* is the average *Network*_*twopathways*_ accuracy obtained using the identity map, the luminance map, or the orientation map.

## Results

Welch's two-sample *t*-tests were used to compare different testing accuracies and determine the significance of the differences. The difference between testing accuracies is considered to be significant if the corresponding *p*-value < 0.05.

### Visual pathway networks

The average testing accuracies of different neural networks for modeling different visual pathways are shown in Table 1A. According to these results, the accuracy of the network was the highest when it was trained to determine an attribute and the map was based on the same attribute (gray cells), or when the map was based on location (yellow cells). For example, the accuracy of *Network*_*identity*_ was the highest when the identity map or the location map was used for training. Similarly, the accuracy of *Network*_*luminance*_ was the highest when the luminance map or the location map was used, the accuracy of *Network*_*orientation*_ was the highest when the orientation map or the location map was used, and the accuracy of *Network*_*location*_ was the highest when the location map was used.

**Table 1:** The original simulation. There are four kinds of objects (t-shirt, pant, shoe, bag).

**(A)**				
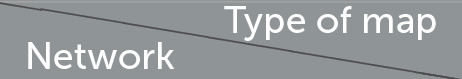	**Identity**	**Luminance**	**Orientation**	**Location**
*Network*_*identity*_	83.1 ± 2.9	47.1 ± 1.3	42.1 ± 3.5	75.7 ± 3.1
*Network*_*luminance*_	36.0 ± 3.2	61.8 ± 3.6	27.3 ± 1.8	61.9 ± 4.6
*Network*_*orientation*_	45.1 ± 2.6	34.7 ± 0.2	85.4 ± 0.8	74.0 ± 1.8
*Network*_*location*_	46.7 ± 0.5	64.9 ± 0.3	59.0 ± 1.3	100.0 ± 0.0
**(B)**				
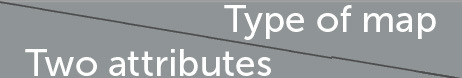	**Identity**	**Luminance**	**Orientation**	**Location**
Identity, luminance	44.6 ± 0.6	42.6 ± 0.4	36.2 ± 0.3	47.1 ± 1.1
Orientation, location	17.7 ± 0.6	15.6 ± 0.4	37.3 ± 0.4	41.2 ± 0.9
Identity, orientation	46.8 ± 0.8	33.4 ± 0.4	49.6 ± 0.7	37.7 ± 0.6
Identity, location	31.9 ± 0.8	26.9 ± 0.5	19.9 ± 1.0	44.8 ± 0.6
Luminance, orientation	20.7 ± 0.5	26.9 ± 1.0	27.6 ± 0.3	35.1 ± 0.5
Luminance, location	19.9 ± 0.2	34.3 ± 0.5	17.0 ± 0.5	42.2 ± 0.5

**(A)** Average testing accuracies in percentage (%) ± standard deviations (%) for networks modeling different visual pathways with different maps. There are four kinds of objects (t-shirt, pant, shoe, bag), four luminance levels, four orientations, and nine locations. The row headers are the names of the networks. The column headers are the types of maps that were used for constraining the binding problem. *Network*_*identity*_, *Network*_*luminance*_, *Network*_*orientation*_, and *Network*_*location*_ were trained to model the identity pathway, the luminance pathway, the orientation pathway, and the location pathway, respectively. The accuracy of the network was the highest when it was trained to determine an attribute and the map was based on the same attribute (gray cells), or when the map was based on location (yellow cells). **(B)** Average testing accuracies in percentage (%) ± standard deviations (%) for different *Network*_*twopathways*_ that were trained to determine different pairs of attributes. There are four kinds of objects, four luminance levels, four orientations, and nine locations. The row headers are the two attributes that *Network*_*twopathways*_ was trained to determine. The column headers are the types of maps that were used for constraining the binding problem. Cells are gray when the network was trained to determine two attributes and the map was based on one of these two attributes, and cells are yellow when the map was based on location. When *Network*_*twopathways*_ was trained to determine identity and orientation, the location map resulted in significantly lower accuracy than the identity map and the orientation map. In all other cases, the location map resulted in significantly higher accuracy.

### Two-pathway networks

The average testing accuracies of different *Network*_*twopathways*_ that were trained to determine different pairs of attributes are shown in Table 1B. According to these results, the accuracy of *Network*_*twopathways*_ was the highest in most cases when the location map was used to constrain the binding problem. For example, even when the two attributes that needed to be determined were identity and luminance, using the location map achieved a significantly higher accuracy than using the identity map or the luminance map. Similar results were also obtained in other situations when the other pairs of attributes were determined using *Network*_*twopathways*_. However, there were exceptions. When *Network*_*twopathways*_ was trained to determine identity and orientation, the accuracy of *Network*_*twopathways*_ with the location map was significantly lower than the accuracy of *Network*_*twopathways*_ with the identity map or the orientation map.

### Why the location map is a better choice than the other maps used in this study

**Figure 3:**
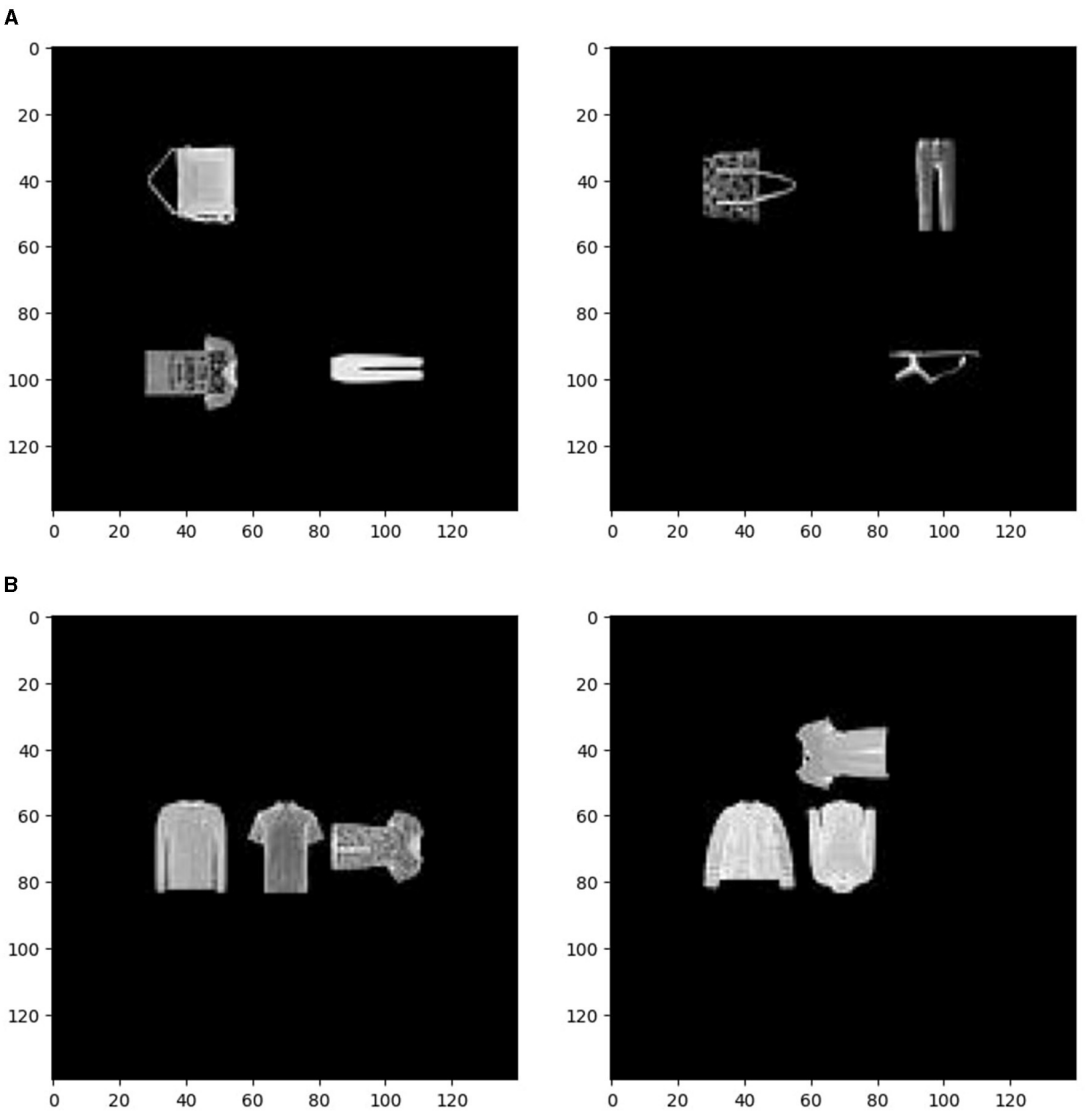
**(A)** Examples of the visual images with four classes in each of the four attributes. **(B)** Examples of the visual images with four kinds of tops: t-shirts, pullovers, coats, and shirts.

To test the hypothesis that the location map is a better choice because there are more classes for location, that is, nine possible locations, but only four possible objects, four possible luminance levels, and four possible orientations, we repeated our experiments with the same number of classes in each of the four attributes. In these simulations, we only kept locations 1, 3, 7, and 9 as the four possible locations. Some examples are shown in Figure 3A. As a result, all four attributes would each have four classes.

The average testing accuracies of different neural networks for modeling different visual pathways, when there were four classes in each attribute, are shown in Table 2A. The average testing accuracies of different *Network*_*twopathways*_ that were trained to determine different pairs of attributes, when there were four classes in each attribute, are shown in Table 2B. According to these results, it was still true that the accuracy of the network was the highest when it was trained to determine an attribute and the map was based on the same attribute, or when the map was based on location. Similarly, the accuracy of *Network*_*twopathways*_ was still the highest when the location map was used, but there were two exceptions. When *Network*_*twopathways*_ was trained to determine identity and orientation, the location map resulted in significantly lower accuracy than the identity map and the orientation map. When *Network*_*twopathways*_ was trained to determine luminance and orientation, the location map resulted in significantly lower accuracy than the luminance map. In all other cases, the location map resulted in significantly higher accuracy. These results indicate that constraining the binding problem using the location map is a better choice in most cases but again there are some exceptions. It is possible that these exceptions may also be caused by the dependency between different attributes.

**Table 2:** The simulation with the same number of classes in each of the four attributes. There are four kinds of objects (t-shirt, pant, shoe, bag).

**(A)**				
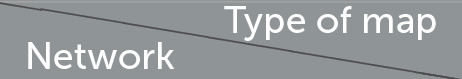	**Identity**	**Luminance**	**Orientation**	**Location**
*Network*_*identity*_	95.9 ± 0.9	66.1 ± 2.2	73.0 ± 1.5	98.8 ± 0.2
*Network*_*luminance*_	59.1 ± 1.0	84.0 ± 0.7	46.7 ± 0.6	86.9 ± 2.9
*Network*_*orientation*_	76.5 ± 2.2	62.4 ± 1.3	97.6 ± 0.6	99.0 ± 0.3
*Network*_*location*_	94.3 ± 0.3	85.8 ± 0.6	95.0 ± 0.7	100.0 ± 0.0
**(B)**				
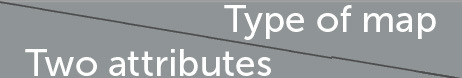	**Identity**	**Luminance**	**Orientation**	**Location**
Identity, luminance	68.7 ± 0.6	70.4 ± 0.4	59.6 ± 0.7	71.9 ± 0.2
Orientation, location	32.0 ± 0.1	32.5 ± 1.4	96.3 ± 0.4	99.4 ± 0.1
Identity, orientation	81.4 ± 0.4	58.1 ± 1.0	77.9 ± 0.9	58.5 ± 0.2
Identity, location	95.1 ± 0.1	63.4 ± 0.9	30.5 ± 0.8	98.4 ± 0.2
Luminance, orientation	40.3 ± 0.9	63.4 ± 1.3	52.7 ± 1.8	56.5 ± 0.4
Luminance, location	53.2 ± 0.5	82.3 ± 0.1	37.1 ± 0.9	82.9 ± 0.1

**(A)** Average testing accuracies in percentage (%) ± standard deviations (%) for networks modeling different visual pathways with different maps. There are four classes in each of the four attributes. There are four kinds of objects (t-shirt, pant, shoe, bag). The row headers are the names of the networks. The column headers are the types of maps that were used for constraining the binding problem. *Network*_*identity*_, *Network*_*luminance*_, *Network*_*orientation*_, and *Network*_*location*_ were trained to model the identity pathway, the luminance pathway, the orientation pathway, and the location pathway, respectively. The accuracy of the network was the highest when it was trained to determine an attribute and the map was based on the same attribute (gray cells), or when the map was based on location (yellow cells). **(B)** Average testing accuracies in percentage (%) ± standard deviations (%) for different *Network*_*twopathways*_ that were trained to determine different pairs of attributes. There are four classes in each of the four attributes. The row headers are the two attributes that *Network*_*twopathways*_ was trained to determine. The column headers are the types of maps that were used for constraining the binding problem. Cells are gray when the network was trained to determine two attributes and the map was based on one of these two attributes, and cells are yellow when the map was based on location. When *Network*_*twopathways*_ was trained to determine identity and orientation, the location map resulted in significantly lower accuracy than the identity map and the orientation map. When *Network*_*twopathways*_ was trained to determine luminance and orientation, the location map resulted in significantly lower accuracy than the luminance map. In all other cases, the location map resulted in significantly higher accuracy.

To test the hypothesis that the location map is better because the objects always have different locations, but may have the same identities, luminance levels, or orientations, we repeated our experiments and ensured that the objects in each visual image always had different identities, luminance levels, orientations, and locations. In these simulations, we used again four possible kinds of objects, four luminance levels, four possible orientations, and nine possible locations. The average testing accuracies of different neural networks for modeling different visual pathways, when the objects always had different identities, luminance levels, orientations, and locations, are shown in Table 3A. The average testing accuracies of different *Network*_*twopathways*_ that were trained to determine different pairs of attributes for these simulations are shown in Table 3B. According to these results, it was still true that the accuracy of the network was the highest when it was trained to determine an attribute and the map was based on the same attribute, or when the map was based on location. The accuracy of *Network*_*twopathways*_ was the highest when the location map was used and there were no exceptions. These results indicate that under the experimental conditions examined in this study, constraining the binding problem using the location map is the best choice regardless of whether the objects' attributes were unique.

**Table 3:** The simulation with all attributes being unique. There are four kinds of objects (t-shirt, pant, shoe, bag).

**(A)**				
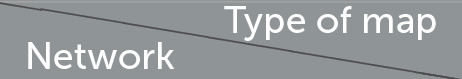	**Identity**	**Luminance**	**Orientation**	**Location**
*Network*_*identity*_	90.3 ± 0.0	76.3 ± 1.6	76.3 ± 1.3	88.7 ± 0.7
*Network*_*luminance*_	65.5 ± 2.2	85.5 ± 0.6	43.7 ± 4.1	80.3 ± 0.7
*Network*_*orientation*_	78.0 ± 3.2	66.7 ± 2.0	90.1 ± 2.7	87.7 ± 0.9
*Network*_*location*_	81.1 ± 2.2	92.3 ± 0.5	87.7 ± 1.2	100.0 ± 0.0
**(B)**				
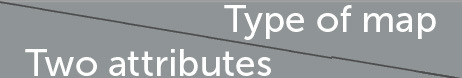	**Identity**	**Luminance**	**Orientation**	**Location**
Identity, luminance	62.8 ± 0.3	66.8 ± 0.3	48.8 ± 0.4	71.8 ± 0.6
Orientation, location	15.7 ± 1.0	21.4 ± 1.0	65.8 ± 1.2	74.8 ± 0.7
Identity, orientation	69.1 ± 0.5	66.1 ± 1.0	73.4 ± 0.6	77.4 ± 0.1
Identity, location	62.3 ± 0.4	49.8 ± 1.1	18.2 ± 0.6	73.2 ± 0.6
Luminance, orientation	51.0 ± 0.3	56.8 ± 0.6	42.5 ± 0.6	71.6 ± 0.4
Luminance, location	29.8 ± 0.8	63.7 ± 1.0	17.3 ± 0.6	69.9 ± 0.9

**(A)** Average testing accuracies in percentage (%) ± standard deviations (%) for networks modeling different visual pathways with different maps. The objects in each visual image always had different identities, luminance levels, orientations, and locations. There are four kinds of objects (t-shirt, pant, shoe, bag), four luminance levels, four orientations, and nine locations. The row headers are the names of the networks. The column headers are the types of maps that were used for constraining the binding problem. *Network*_*identity*_, *Network*_*luminance*_, *Network*_*orientation*_, and *Network*_*location*_ were trained to model the identity pathway, the luminance pathway, the orientation pathway, and the location pathway, respectively. The accuracy of the network was the highest when it was trained to determine an attribute and the map was based on the same attribute (gray cells), or when the map was based on location (yellow cells). **(B)** Average testing accuracies in percentage (%) ± standard deviations (%) for different *Network*_*twopathways*_ that were trained to determine different pairs of attributes. The objects in each visual image always had different identities, luminance levels, orientations, and locations. There are four kinds of objects, four luminance levels, four orientations, and nine locations. The row headers are the two attributes that *Network*_*twopathways*_ was trained to determine. The column headers are the types of maps that were used for constraining the binding problem. Cells are gray when the network was trained to determine two attributes and the map was based on one of these two attributes, and cells are yellow when the map was based on location. In all cases, the location map resulted in significantly higher accuracy.

### The exceptions mentioned above may be caused by the dependencies between orientation, luminance, and identity

It is possible that there are dependencies between orientation, luminance, and identity and that these dependencies may cause the exceptions in our results.

To determine the orientations of t-shirts, pants, shoes, and bags, the neural network could use different ways (different kinds of features). For example, the orientations of t-shirts may be primarily dependent on the places of the sleeves whereas the orientations of bags may be primarily dependent on the places of the handles. Hence, it is possible that the identity information contained in the identity map may be helpful for the orientation task. Likewise, because the orientation map was obtained based on the orientation task, it is possible that the orientation map contains identity information. Therefore, it is possible that the identity information in the orientation map may also help the network complete the identity task. As a result, the neural network would be able to determine identity and orientation with higher accuracy when the identity map or the orientation map was used.

In addition, t-shirts, pants, shoes, and bags have different luminance. The average luminance of shoes (33.6 ± 14.9) was significantly lower than the average luminance of pants (57.2 ± 13.7). The average luminance of pants (57.2 ± 13.7) was significantly lower than the average luminance of t-shirts (81.0 ± 28.6) and bags (85.8 ± 26.2). Therefore, the neural network may also retain identity information in the luminance map and this identity information would be helpful for the orientation task. However, the orientation map may not contain information about luminance since the orientation task is not directly dependent on luminance. As a result, the neural network would be able to determine luminance and orientation with higher accuracy only when the luminance map was used.

We conducted additional simulations to test these hypotheses. We removed the dependency between orientation, luminance, and identity by using similar kinds of objects in our input images. We used 200 t-shirts, 200 pullovers, 200 coats, and 200 shirts as the four kinds of objects. Then, we created the input images in the same way as before. Though we still have four kinds of objects, all of them are tops. Therefore, in this case, the neural network should use almost the same way (same kinds of features) to determine the orientations of objects. As a result, the identity information should no longer be helpful for the orientation task. In addition, the four kinds of tops have relatively similar luminance (t-shirts: 81.0 ± 28.6, pullovers: 95.9 ± 33.2, coats: 100.9 ± 26.4, shirts: 86.9 ± 33.3), so now identity should also be almost independent of luminance. Two example visual images are shown in Figure 3B.

We repeated the three kinds of simulations that we did above with the new objects. The results are shown in Tables 4A, B–6A, B. To make the accuracies easier to compare, a larger dataset (12,000 visual images) was used to obtain the results in Tables 4A, B. Datasets with the same size as before (six thousand visual images) were used to obtain the results in the other tables.

**Table 4:** The original simulation. There are four kinds of tops (t-shirt, pullover, coat, shirt).

**(A)**				
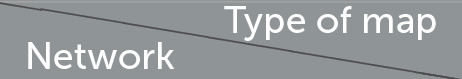	**Identity**	**Luminance**	**Orientation**	**Location**
*Network*_*identity*_	33.9 ± 0.2	13.3 ± 0.8	12.4 ± 1.4	30.3 ± 5.1
*Network*_*luminance*_	35.6 ± 3.0	79.9 ± 3.2	46.7 ± 0.8	82.8 ± 2.1
*Network*_*orientation*_	45.4 ± 0.8	66.5 ± 0.9	97.3 ± 0.5	96.4 ± 0.4
*Network*_*location*_	42.6 ± 3.4	80.6 ± 1.4	85.8 ± 1.4	100.0 ± 0.0
**(B)**				
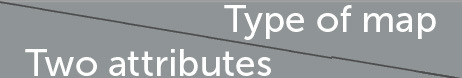	**Identity**	**Luminance**	**Orientation**	**Location**
Identity, luminance	15.3 ± 0.4	12.3 ± 0.1	11.4 ± 0.6	22.8 ± 0.3
Orientation, location	29.1 ± 1.2	28.8 ± 0.9	70.9 ± 0.8	74.8 ± 0.3
Identity, orientation	18.6 ± 0.3	11.4 ± 0.1	14.0 ± 0.2	24.8 ± 0.7
Identity, location	15.7 ± 0.4	7.2 ± 0.1	6.6 ± 0.5	23.7 ± 0.8
Luminance, orientation	40.6 ± 0.8	59.9 ± 0.3	52.5 ± 0.5	75.6 ± 0.3
Luminance, location	29.5 ± 0.2	62.8 ± 0.2	29.4 ± 0.4	75.5 ± 0.4

**(A)** Average testing accuracies in percentage (%) ± standard deviations (%) for networks modeling different visual pathways with different maps. There are four kinds of tops (t-shirt, pullover, coat, shirt), four luminance levels, four orientations, and nine locations. The row headers are the names of the networks. The column headers are the types of maps that were used for constraining the binding problem. *Network*_*identity*_, *Network*_*luminance*_, *Network*_*orientation*_, and *Network*_*location*_ were trained to model the identity pathway, the luminance pathway, the orientation pathway, and the location pathway, respectively. The accuracy of the network was the highest when it was trained to determine an attribute and the map was based on the same attribute (gray cells), or when the map was based on location (yellow cells). **(B)** Average testing accuracies in percentage (%) ± standard deviations (%) for different *Network*_*twopathways*_ that were trained to determine different pairs of attributes. There are four kinds of tops, four luminance levels, four orientations, and nine locations. The row headers are the two attributes that *Network*_*twopathways*_ was trained to determine. The column headers are the types of maps that were used for constraining the binding problem. Cells are gray when the network was trained to determine two attributes and the map was based on one of these two attributes, and cells are yellow when the map was based on location. In all cases, the location map resulted in significantly higher accuracy.

**Table 5:** The simulation with the same number of classes in each of the four attributes. There are four kinds of tops (t-shirt, pullover, coat, shirt).

**(A)**				
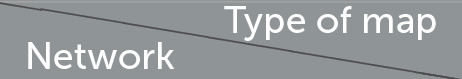	**Identity**	**Luminance**	**Orientation**	**Location**
*Network*_*identity*_	51.1 ± 1.3	19.1 ± 0.6	14.8 ± 1.4	71.8 ± 4.6
*Network*_*luminance*_	45.8 ± 1.0	89.8 ± 2.1	57.5 ± 1.7	94.7 ± 1.0
*Network*_*orientation*_	50.3 ± 0.6	66.7 ± 1.3	97.9 ± 0.1	98.3 ± 0.3
*Network*_*location*_	72.1 ± 1.8	88.8 ± 0.9	97.1 ± 0.2	100.0 ± 0.0
**(B)**				
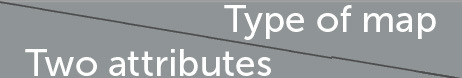	**Identity**	**Luminance**	**Orientation**	**Location**
Identity, luminance	28.7 ± 0.5	20.9 ± 0.5	16.6 ± 0.4	57.3 ± 0.4
Orientation, location	34.8 ± 1.8	34.3 ± 1.7	97.9 ± 0.3	98.8 ± 0.0
Identity, orientation	33.2 ± 0.4	15.5 ± 1.0	17.0 ± 0.4	46.2 ± 0.6
Identity, location	51.1 ± 0.2	15.0 ± 0.3	9.3 ± 0.7	73.4 ± 0.7
Luminance, orientation	46.0 ± 1.5	68.8 ± 0.8	65.9 ± 0.3	77.7 ± 0.3
Luminance, location	50.1 ± 0.9	88.1 ± 0.5	67.6 ± 1.1	94.2 ± 0.4

**(A)** Average testing accuracies in percentage (%) ± standard deviations (%) for networks modeling different visual pathways with different maps. There are four classes in each of the four attributes. There are four kinds of tops (t-shirt, pullover, coat, shirt). The row headers are the names of the networks. The column headers are the types of maps that were used for constraining the binding problem. *Network*_*identity*_, *Network*_*luminance*_, *Network*_*orientation*_, and *Network*_*location*_ were trained to model the identity pathway, the luminance pathway, the orientation pathway, and the location pathway, respectively. The accuracy of the network was the highest when it was trained to determine an attribute and the map was based on the same attribute (gray cells), or when the map was based on location (yellow cells). **(B)** Average testing accuracies in percentage (%) ± standard deviations (%) for different *Network*_*twopathways*_ that were trained to determine different pairs of attributes. There are four classes in each of the four attributes. The row headers are the two attributes that *Network*_*twopathways*_ was trained to determine. The column headers are the types of maps that were used for constraining the binding problem. Cells are gray when the network was trained to determine two attributes and the map was based on one of these two attributes, and cells are yellow when the map was based on location. In all cases, the location map resulted in significantly higher accuracy.

**Table 6:** The simulation with all attributes being unique. There are four kinds of tops (t-shirt, pullover, coat, shirt).

**(A)**				
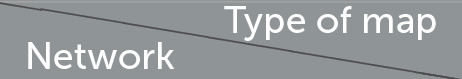	**Identity**	**Luminance**	**Orientation**	**Location**
*Network*_*identity*_	46.7 ± 1.7	13.2 ± 1.3	17.6 ± 1.2	32.2 ± 1.7
*Network*_*luminance*_	29.5 ± 0.9	94.7 ± 0.6	49.8 ± 1.1	89.9 ± 1.8
*Network*_*orientation*_	35.6 ± 0.7	67.9 ± 2.0	91.6 ± 0.4	88.2 ± 2.1
*Network*_*location*_	41.2 ± 2.1	93.9 ± 0.8	90.5 ± 1.8	100.0 ± 0.0
**(B)**				
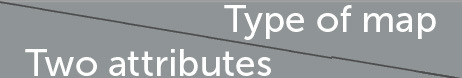	**Identity**	**Luminance**	**Orientation**	**Location**
Identity, luminance	14.1 ± 0.2	12.1 ± 0.4	15.4 ± 0.7	28.8 ± 0.7
Orientation, location	15.7 ± 0.7	23.2 ± 1.0	72.1 ± 0.6	77.1 ± 0.4
Identity, orientation	18.1 ± 0.3	12.1 ± 0.5	16.8 ± 0.3	27.8 ± 0.7
Identity, location	16.4 ± 0.8	8.7 ± 0.2	7.7 ± 0.3	30.2 ± 0.6
Luminance, orientation	21.8 ± 0.5	66.7 ± 0.4	48.0 ± 0.3	75.2 ± 0.5
Luminance, location	19.6 ± 1.5	74.6 ± 0.7	16.9 ± 1.0	77.4 ± 0.3

**(A)** Average testing accuracies in percentage (%) ± standard deviations (%) for networks modeling different visual pathways with different maps. The objects in each visual image always had different identities, luminance levels, orientations, and locations. There are four kinds of tops (t-shirt, pullover, coat, shirt), four luminance levels, four orientations, and nine locations. The row headers are the names of the networks. The column headers are the types of maps that were used for constraining the binding problem. *Network*_*identity*_, *Network*_*luminance*_, *Network*_*orientation*_, and *Network*_*location*_ were trained to model the identity pathway, the luminance pathway, the orientation pathway, and the location pathway, respectively. The accuracy of the network was the highest when it was trained to determine an attribute and the map was based on the same attribute (gray cells), or when the map was based on location (yellow cells). **(B)** Average testing accuracies in percentage (%) ± standard deviations (%) for different *Network*_*twopathways*_ that were trained to determine different pairs of attributes. The objects in each visual image always had different identities, luminance levels, orientations, and locations. There are four kinds of tops, four luminance levels, four orientations, and nine locations. The row headers are the two attributes that *Network*_*twopathways*_ was trained to determine. The column headers are the types of maps that were used for constraining the binding problem. Cells are gray when the network was trained to determine two attributes and the map was based on one of these two attributes, and cells are yellow when the map was based on location. In all cases, the location map resulted in significantly higher accuracy.

**Figure 4:**
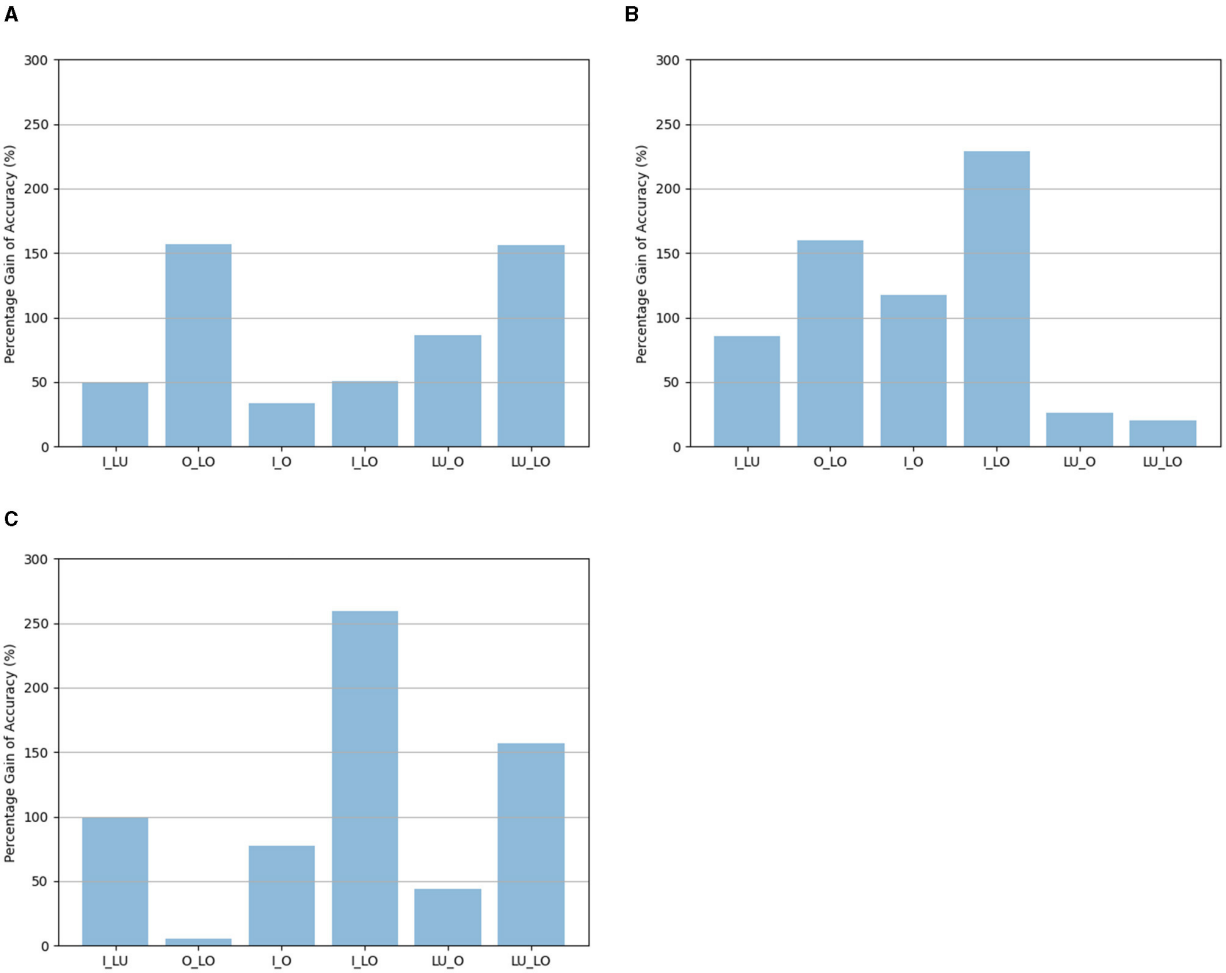
**(A)** The percentage gains of accuracy when the location map was used compared to the identity map. **(B)** The percentage gains of accuracy when the location map was used compared to the luminance map. **(C)** The percentage gains of accuracy when the location map was used compared to the orientation map. The percentage gains of accuracy were calculated by $Gain=\frac{Location\;Map\;Accuracy-Other\;Map\;Accuracy}{Other\;Map\;Accuracy}\times 100\%$. Where *LocationMapAccuracy* is the average *Network*_*twopathways*_ accuracy obtained using the location map, *OtherMapAccuracy* is the average *Network*_*twopathways*_ accuracy obtained using the identity map, the luminance map, or the orientation map. The horizontal labels represent the tasks: I_LU is recognizing identity and luminance, O_LO is recognizing orientation and location, I_O is recognizing identity and orientation, I_LO is recognizing identity and location, LU_O is recognizing luminance and orientation, LU_LO is recognizing luminance and location.

According to the results, the accuracy of the visual pathway network was still the highest when it was trained to determine an attribute and the map was based on the same attribute, or when the map was based on location. More importantly, in all cases, the accuracy of *Network*_*twopathways*_ was always the highest when the location map was used. After removing the dependency between different attributes, the location map is a better choice than the other maps, without exception in the current study, for constraining the binding problem. After removing the dependency between different attributes, the percentage gains of accuracy for different tasks when the location map was used compared to the identity map, the luminance map, or the orientation map are shown in Figure 4. The percentage gains of accuracy in Figure 4 are calculated based on the data in Table 4.

## Discussion

Previous studies have shown it is advantageous to use two-pathway networks as opposed to one-pathway networks to bind two attributes of an object in a multi-object display using a location map (Han and Sereno, 2023a). In our current study, we generalized our findings of multiple-object displays to include four possible attributes of the objects (identity, luminance, orientation, and location) and explored which kind of map is the better choice for constraining the binding problem. We found that for any pairs of two attributes examined in this study, which were independent of each other, that the two-pathway network needed to recognize and bind, it was always better in these experiments to use the location map to constrain the binding problem to achieve the highest accuracy. We also showed that under the conditions examined here and when visual attributes were independent of each other, the advantage of using the location map was independent of the number of classes in each attribute and independent of whether the objects could share the same value in a particular attribute. As clarified in the introduction, any usage or claim of “best” or “optimal” or “always” is limited to the conditions and simulations we have conducted in our current study.

### A location map is not always a better choice if there are dependencies between different attributes

According to our simulations, the location map did not always result in the highest accuracy when there were dependencies between orientation, luminance, and identity. In Tables 1B, 2B, the orientation map and the identity map resulted in significantly higher accuracy than the location map when *Network*_*twopathways*_ was trained to determine identity and orientation. Also in Table 2B, the luminance map resulted in significantly higher accuracy than the location map when *Network*_*twopathways*_ was trained to determine luminance and orientation. However, these exceptions might be caused by the interactions between different visual attributes. For example, the neural networks might use different ways to determine the orientations of t-shirts, pants, shoes, and bags. As a result, there might be interactions between orientation and identity and these interactions might cause the exception that using an identity map or orientation map could achieve higher accuracy than using a location map. All of the exceptions disappeared after we removed the dependencies by using four kinds of tops as the different objects in the input images. It suggests that the spatial location map is not always better when there are dependencies between different attributes. Interestingly, perhaps in agreement with these findings, previous study in primates suggests that there are more complex (e.g., combined attribute) selectivities in higher visual areas (Tanaka, 2003) as well as evidence that there are less clear spatial maps (retinotopy) in these higher visual areas (e.g., Rajimehr et al., 2014).

### When different attributes are independent, the spatial location map is a better choice for binding in two-pathway networks, and maps based on a trained attribute are not better choices for visual binding

After removing dependencies between different attributes, the accuracy of *Network*_*twopathways*_ in these experiments was always the highest when the location map was used to constrain the binding problem, no matter which two attributes that *Network*_*twopathways*_ was trained to recognize. These results are interesting because in our previous study, we have shown that when *Network*_*twopathways*_ was trained to determine identity and location, it was able to associate each object's identity with its location using the related but differently retained location information in the two pathways (relative location information in the identity pathway and absolute location information in the location pathway). Intuitively, one might think that *Network*_*twopathways*_ would have a similar performance when it was trained to use the related but differently retained identity information in the two pathways to associate each object's identity with its location. In addition, one might think when *Network*_*twopathways*_ needs to determine other attributes, for example, identity and luminance, it would be better to associate each object's identity and luminance using the related but differently retained identity or luminance information in the two pathways. However, the simulation results in our current study showed that *Network*_*twopathways*_ associated any two attributes of each object together better by using the related but differently retained location information in the two pathways, even when location was not one of the two attributes that was trained to be recognized by either visual pathway network contained in *Network*_*twopathways*_. In other words, in these experiments, it was always better to use a location map to constrain the binding problem when there is not dependency between different attributes.

### A location map better for visual binding—neurophysiological evidence

Our study indicates that to process multiple attributes of multiple objects in parallel segregated pathways and then bind them together, it is better for the brain to use a spatial map to encode the attributes of objects. Many experimental studies have shown that there are topographic maps in the brain and that these maps in the visual system are primarily spatial (Kaas, 1997; Lennie, 1998). For example, the orientation “pinwheels” in primates and cats representing different orientations are arranged radially around a central location with all orientations represented in every patch of the retinotopic map (Ibbotson and Jung, 2020). These orientation “pinwheels” at different places in the retinotopic map suggest that orientation is embedded in a location map. Our study, using a computational approach, shows that a location map is computationally advantageous to bind multiple attributes of multiple visual objects, perhaps providing insight into the topography of visual cortical areas.

### A location map for visual binding—mechanistic models

Many previous modeling studies, using more mechanistic models, have also used spatially organized maps to bind different kinds of visual information together. For example, studies conducted by Layton et al. (2012, 2014) and Layton and Yazdanbakhsh (2015) used spatial maps in their computational models at the level of microcircuits to bind border ownership, fragmented pieces of orientation, and camouflage pieces to explain how the visual system performs figure-ground segregation. In addition, Zhou et al. (2000) used spatially organized models of early visual areas to bind different visual information and explain the coding of border ownership in the brain. Park et al. (2022) studied the impact of orientation interactions on visual illusions using a model with orientation information encoded within a retinotopic location map. The common use of spatial maps in these studies suggest that spatial maps may be a propitious choice for constraining the binding problem.

The retinotopic maps in the brain approximate logarithmic maps, not Cartesian maps (Wu et al., 2012). The location map used in our study is not a realistic retinotopic map (Ta et al., 2022). However, because we use a relative location map in our model (e.g., object A is on the top left of object B), the specific layout (Cartesian, logarithmic, or based on the Human Connectome Project) should not be important for our model to work, that is, our model suggests that the relative location information in the retinotopic maps is enough to constrain the binding problem.

### Why is a location map better for visual binding?

Though we have shown that it is computationally advantageous to embed feature maps in spatial location in the visual system, it is unclear why the location map is better than other kinds of maps in the visual system.

One possible reason for the spatial location map being a better choice is that there are nine possible locations, but only four possible identities, four possible luminance levels, and four possible orientations for each object. With more number of possible locations, the neural network may be able to differentiate different objects better according to their locations and then bind multiple attributes together. To test this hypothesis, we did additional simulations with the same number of classes in each attribute. We ensured that each object had four possible identities, four possible luminance levels, four possible orientations, and four possible locations. According to these simulations, *Network*_*twopathways*_ still achieved the highest accuracy with the location map. These results indicate that the location map is the better choice in this study regardless of the number of classes in each attribute.

Another possible reason for the spatial location map being a better choice is that in our study, we assumed the locations of objects are always different, but the identities, luminance, and orientations of different objects could be the same. Though the assumption is artificial, it generally agrees with real life experience, that is, the locations of multiple objects are typically different, but the other attributes of the objects may be repeated (the same). The unique location of each object may provide a better “clustered index” that can be used to bind multiple attributes together. To test the importance of uniqueness, we did additional simulations and ensured that all objects in the same visual image also never had the same identities, luminance levels, or orientations. According to these simulations, *Network*_*twopathways*_ also achieved the highest accuracy with the location map. These results indicate that under the conditions that we examine, the location map is the best choice even when the other attributes are also unique.

Finally, it is possible that location might align better between the world and the visual sensory epithelium, so it is better to use a location map. Though sometimes refraction, reflection, and diffraction could change the direction of light, light travels along straight lines in most cases so that the light that comes from objects at different locations will naturally arrive at different corresponding locations on the retina. The brain can easily retain this information with retinotopic mapping. However, it might be more difficult for the visual system to determine identity, luminance, and orientation of an object and hence would require more complex information processing.

### What is a better map for binding in other sensory systems?

Though we have shown that it is computationally advantageous to embed feature maps in spatial location in the visual system, it is unclear whether this is also the better choice for other sensory systems. Some studies have shown that some other sensory systems do not primarily use a spatial map. For example, tonotopic maps of sound frequency have been documented in cortical auditory cortex (Talavage et al., 2000) and maps of odors based on features in chemical space in the olfactory system (Imai et al., 2010). Furthermore, in auditory cortex, no explicit cortical spatial map of sound localization has been found, despite 40 years of searching (Middlebrooks, 2021). Nevertheless, spatial features of auditory cortex such as panoramic spatial coding of single cell responses, task-dependent sharpening of spatial sensitivity, and direct projections to space-mapped brain regions (e.g., parietal visual cortical areas and subcortical oculomotor structures such as the superior colliculus) suggest some sort of distributed code for sound-source location likely exists in auditory cortex (for review, see Middlebrooks, 2021). The fact that different attributes define the primary topology in different sensory systems suggests that the location map may not be the only better choice for all sensory systems and that it is possible that the other sensory systems might use maps based on other attributes to constrain the binding problem.

Coding of specific attributes may differ in difficulty for different sensory systems. For example, it might be the easiest for the visual system to determine location because light travels along straight lines in most cases, but it is not the case for sound waves. Sound waves have much longer wavelength and usually do not travel along straight lines because of diffraction. It might be easiest for the human auditory system to determine sound frequency as opposed to spatial location because the auditory sensory epithelium is laid out by frequency. Similarly, it might also be easiest for the olfactory system to determine features of chemical space as opposed to spatial location. Therefore, the auditory system and the olfactory system may bind attributes of auditory or olfactory signals primarily using a frequency map and a chemical space map, respectively. Furthermore, specific attributes may differ in importance for different sensory systems. Perhaps, sound frequency or odor is more important than spatial localization in auditory processing or olfaction, respectively. However, as these sensory systems all need to interact with spatially mapped motor output, it may be that space is helpful to align the maps (Rees, 1996). Future work is needed to clarify these speculations.

## Limitations and future directions

One limitation of our study is that to control conditions we used relatively simple images and models to test our hypotheses in simple artificial settings. In future, it would be interesting to see whether these findings would be similar for more complex natural images, for visual tasks that involve more visual attributes, and for models that recognize orientations and locations of objects across a continuum.

In addition, in future, it would be interesting to test whether the location map could improve not only the accuracy but also the efficiency of the network. For example, in a noisy environment, whether the network could recognize and associate objects' visual attributes faster using the location map.

## Conclusion

Previous work has shown that it is advantageous (both in accuracy and efficiency) to process attributes of objects using artificial neural networks with two segregated pathways as opposed to a single pathway. However, when using such artificial neural networks to process and bind multiple objects' attributes, there is a binding problem, given that segregated pathways may not recombine and associate each object's attributes appropriately. In a previous study, we successfully constrained the binding problem by using a relative location map, but theoretically maps based on other attributes should work as well. Here, we compare the performance of the networks using maps based on different attributes (identity, luminance, orientation, and location) to find out which kind of map is a better choice for constraining the binding problem. Although using a map based on a given trained attribute of a network could be successful, we found that in our experiments, the location map was always a better choice than other maps considered in this study to constrain the binding problem when attributes were independent. We also found that when attributes were independent, the location map was a better choice regardless of the number of classes in each attribute and whether the objects in the visual image could have the same value in each attribute. Though our results agree with the previous neurophysiological findings that show that the map in many visual cortical areas is primarily spatial, the maps in other sensory systems are not always primarily spatial. The underlying general principle for choosing the better map in different sensory systems requires future investigations.

## Data availability statement

The raw data supporting the conclusions of this article may be made available to qualified researchers on reasonable request to the corresponding author.

## Author contributions

ZH: Conceptualization, Data curation, Formal analysis, Investigation, Methodology, Software, Visualization, Writing – original draft, Writing – review & editing. AS: Conceptualization, Funding acquisition, Supervision, Writing – review & editing.
